# Pharmacokinetics and Nephrotoxicity of Polymyxin MRX-8 in Rats: A Novel Agent against Resistant Gram-Negative Bacteria

**DOI:** 10.3390/antibiotics13040354

**Published:** 2024-04-12

**Authors:** Xingyi Qu, Chenxue Guo, Shaojun Liu, Xin Li, Lin Xi, Xiaofen Liu, Jing Zhang

**Affiliations:** 1 Institute of Antibiotics, Huashan Hospital, Fudan University, Shanghai 200040, China; 20111030085@fudan.edu.cn (X.Q.); 20211220020@fudan.edu.cn (C.G.); lixin@huashan.org.cn (X.L.); 22211220011@m.fudan.edu.cn (L.X.); 2 Key Laboratory of Clinical Pharmacology of Antibiotics, Shanghai 200040, China; 3 National Health Commission & National Clinical Research Center for Aging and Medicine, Huashan Hospital, Fudan University, Shanghai 200040, China; 4 Division of Nephrology, Huashan Hospital, Fudan University, Shanghai 200052, China; liushaojun@fudan.edu.cn; 5 Clinical Pharmacology Center, Huashan Hospital, Fudan University, Shanghai 200437, China

**Keywords:** MRX-8, polymyxins, pharmacokinetics, nephrotoxicity, MRX-8039

## Abstract

MRX-8 is a novel polymyxin for carbapenem-resistant Gram-negative infections that has been recently evaluated in Phase I clinical trials. Herein, its pharmacokinetics (PK) and nephrotoxicity in rats are reported for the first time. This study aimed at pre-clinical PK and safety assessments. An LC-MS/MS method was developed to determine concentrations of MRX-8 and its major deacylation metabolite, MRX-8039, in rat plasma. Animals were administered a single dose of MRX-8 (2, 4, 6, and 8 mg/kg) or comparator polymyxin B (PMB) (4 and 8 mg/kg) to compare the kidney injury known for the polymyxin drug class. Nephrotoxicity was evaluated using serum creatinine, blood urea nitrogen (BUN) biomarkers, and renal histopathology. In rats, MRX-8 displayed linear PK within the range of 2–8 mg/kg, with approximately 4% of MRX-8 converted to MRX-8039. MRX-8 induced only mild increases in serum creatinine and BUN levels, with an apparent decrease in nephrotoxicity within 24 h, in contrast to PMB, which exhibited a significant and more persistent toxicity. Additional nephrotoxicity biomarkers (plasma NGAL and urinary NGAL, KIM-1, and TIMP-1) have confirmed attenuated MRX-8 kidney injury. Histopathology has revealed significantly greater cellular/tissue toxicity for PMB as compared to MRX-8 (variances of *p* = 0.008 and *p* = 0.048 vs. saline control, respectively). Thus, MRX-8 induces a mild and reversible kidney injury in rats compared to PMB. These data support a continued evaluation of the novel polymyxin in human trials.

## 1. Introduction

The incidence of infections stemming from multidrug-resistant Gram-negative bacteria is rising, presenting a formidable challenge [1]. Antimicrobial resistance contributes to heightened complexity in infection management and an elevated mortality risk [2,3]. In light of the favorable microbial susceptibility of the polymyxin agents, this drug class is currently being reassessed and reintroduced for critical therapeutic applications [4]. However, nephrotoxicity and neurotoxicity were the most common adverse reactions associated with polymyxins, which limited the clinical dosage [5]. The occurrence of nephrotoxicity, typically manifested as acute kidney injury (AKI), limits its utility [6] and may result in altered pharmacokinetics of concurrently administered drugs, complicating the clinical treatment of critically ill patients [7]. Hence, there is an urgent need for novel polymyxin agents with lower toxicity.

**Figure 1:**
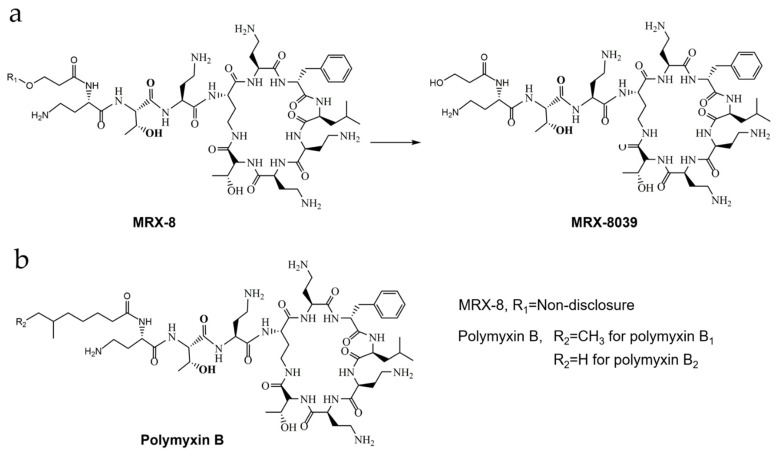
Structures of MRX-8, metabolite MRX-8039, and polymyxin B. (**a**) Chemical structures and transformation of MRX-8 and MRX-8039; (**b**) Chemical structure of polymyxin B.

The next-generation polymyxin agent MRX-8 was introduced to address nephrotoxicity concerns through a “soft drug design” [8,9]. As reported, MRX-8 has the potential to form a major metabolite, MRX-8039, through the hydrolysis of a designer ester bond at the N-Terminus side chain [9,10]. This metabolite purports to exhibit reduced nephrotoxicity as compared to the drug MRX-8. The structures of MRX-8, MRX-8039, and polymyxin B (PMB) are shown in Figure 1 [10]. Two *in vitro* investigations assessed the pharmacological efficacy of MRX-8 against clinical isolates collected between 2017 and 2020 in China and the US [9,11]. MRX-8 exhibited remarkable potency with MIC_50_ and MIC_90_ values equal to or less than PMB, with no appreciable bacterial resistance. The PK/PD investigations in neutropenic mouse models revealed linear plasma pharmacokinetics for MRX-8, with efficacy strongly associated with C_max_ (maximum concentration of drug)/MIC and AUC (area under the drug concentration–time curve)/MIC ratios [8]. MRX-8 demonstrated superior efficacy compared to PMB, particularly against *Pseudomonas aeruginosa* and *Acinetobacter baumannii* in thigh and lung models, showcasing its potential as a promising polymyxin analog.

In this study, we have developed analytical methods to determine levels of MRX-8 and its metabolite MRX-8039 in rat plasma, as well as their pharmacokinetics and nephrotoxicity in rats. Specifically, we have established and validated LC-MS/MS methods for MRX-8 and MRX-8039 and further assessed the pharmacokinetics and renal safety profile of MRX-8 in rats. This study aims to support the dosage regimen design for subsequent clinical trials of MRX-8.

## 2. Results

### 2.1. LC-MS/MS Method Development for MRX-8 and Metabolite MRX-8039

**Figure 2:**
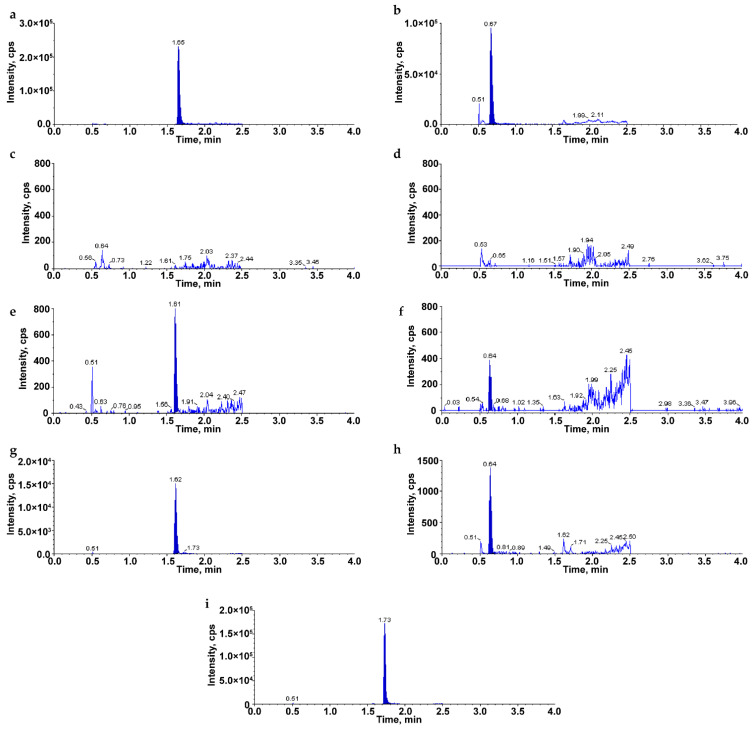
Typical chromatograms of MRX-8, MRX-8039, and IS spiked in rat plasma. (**a**) Chromatogram of MRX-8 under parent ion monitoring mode; (**b**) chromatogram of MRX-8039 under parent ion monitoring mode; (**c**) chromatogram of MRX-8 in blank plasma without spiking MRX-8 and MRX-8039; (**d**) chromatogram of MRX-8039 in blank plasma without spiking MRX-8 and MRX-8039; (**e**) chromatogram of MRX-8 in plasma at LLOQ level; (**f**) chromatogram of MRX-8039 in plasma at LLOQ level; (**g**) chromatogram of MRX-8 in plasma sample after subcutaneous administration of MRX-8; (**h**) chromatogram of MRX-8039 in plasma sample after subcutaneous administration of MRX-8; (**i**) chromatogram of IS spiked in plasma (20 mg/L).

Optimizing the multiple reaction monitoring (MRM) ion pairs involved infusing solutions (20% acetonitrile containing 0.1% formic acid) of MRX-8 and its primary metabolite, MRX-8039, into the mass spectrometry system via a syringe pump. The mass-to-doubly charge ratio (*m*/*z*) values of product ion/daughter ion pairs for ionized [M + 2H]^2+^ and alike species were determined as 617.4/155.1 for MRX-8 and 568.4/173.1 for MRX-8039 (Figure S1). The traces of MRX-8 and MRX-8039 divalent parent ion chromatograms are shown in Figure 2a,b. Additionally, the *m*/*z* was established as 402.3/101.2 for internal standard (IS) polymyxin B1, as reported previously [12].

The mobile phase composition, including the organic phase of acetonitrile, yielded a higher response than methanol and was optimized toward the quantitation of the drug MRX-8 and its metabolite. The addition of 0.1% formic acid to the mobile phase improved both the chromatographic peak response and symmetry. In addition, to minimize the cleavage of the ester MRX-8 into the related alcohol MRX-8039 in the ESI spray ion source, ammonium acetate was added to the aqueous phase. Testing the concentrations of ammonium acetate from 1 mM to 5 mM, 2 mM ammonium acetate significantly reduced the intra-source cleavage, and increased peak area by ~140% for MRX-8 and ~120% for MRX-8039, respectively. As a result, a solution containing 2 mM ammonium acetate and 0.1% formic acid was adopted as the aqueous mobile phase.

### 2.2. LC-MS/MS Method Validation

The selectivity of the LC-MS/MS was evaluated using blank rat plasma from six individuals. Figure 2c–i displays chromatograms from samples of blank plasma, spiking lower limit of quantitation (LLOQ) concentrations of MRX-8 and MRX-8039 in plasma, and typical plasma samples after the administration of MRX-8 in rats. The retention times of compounds MRX-8, MRX-8039, and IS were ~1.62, 0.64, and 1.73 min, respectively. The blank plasma samples showed there was no interference from the plasma matrix. Peak area response ratios of MRX-8 or MRX-8039 to the IS in rat plasma correlated linearly with their concentrations, demonstrating a strong correlation coefficient of (R^2^ > 0.99) within the calibration curve ranges of 0.0100 to 10.0 mg/L for both analytes. The typical calibration equations of calibration curves were y = 1.0446x − 0.0069 for MRX-8 (R^2^ = 0.9963) and y = 1.000x + 0.0233 for MRX-8039 (R^2^ = 0.9965), respectively. The LLOQ concentrations of MRX-8 and MRX-8039 accorded with the signal-to-noise ratio ≥5, with good accuracy of the bias ≤ 20% (MRX-8 from −11.1% to 16.4%, and MRX-8039 from −17.8 to 18.2%), and good precision of relative standard deviation ≤ 20% (MRX-8 from 3.4% to 7.8%, and MRX-8039 from 8.0% to 18.1%). Table 1 summarizes the intra- and inter-day accuracy and precision at four concentration levels of LLOQ, quality control samples of low, medium, and high (QCL, QCM, and QCH). Table S2 summarizes recovery and matrix effect assessments for MRX-8 and MRX-8039 in rat plasma. MRX-8 showed recovery rates ranging from 87.8% to 97.7%, while MRX-8039 exhibited rates between 85.1% and 93.9%. Table S3 shows the stability of both compounds under freeze–thaw cycles and short-term and long-term storage conditions. The established LC-MS/MS method could reliably, accurately, and precisely quantify the concentrations of MRX-8 and MRX-8039 in rat plasma and apply to pharmacokinetic studies in rats.

### 2.3. Pharmacokinetics of MRX-8 and PMB in Rats

**Figure 3:**
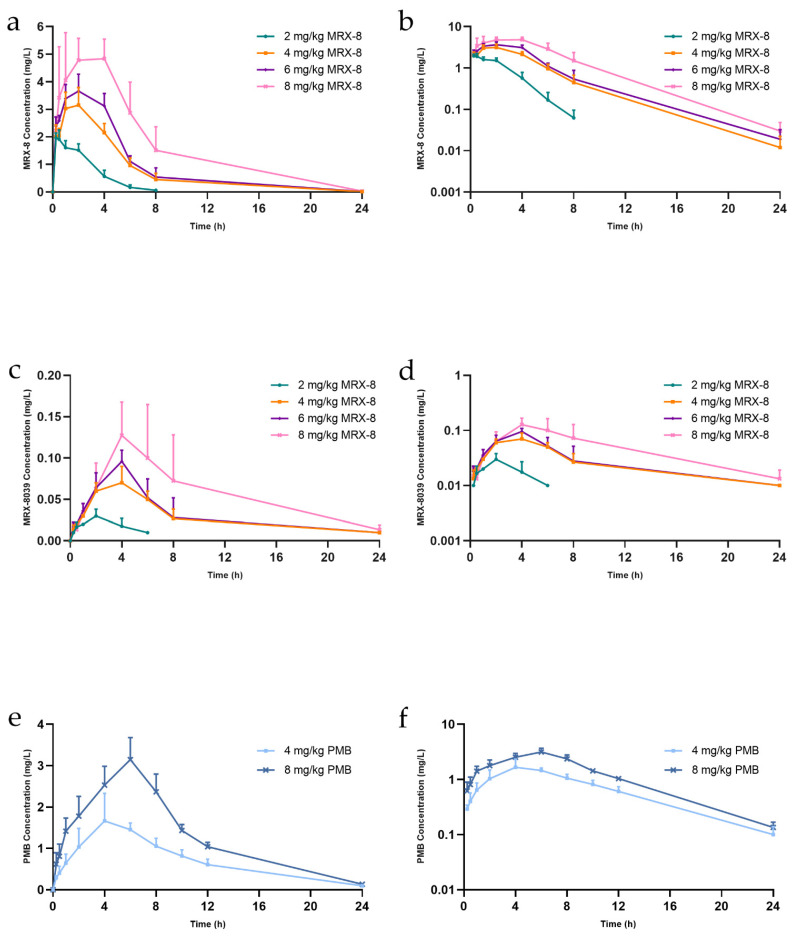
The concentration–time curves of MRX-8, MRX-8039, and PMB in rat plasma after subcutaneous administration. (**a**) Concentration–time curves of MRX-8. (**b**) Semi-logarithmic concentration–time curves of MRX-8. (**c**) Concentration–time curves of MRX-8039. (**d**) Semi-logarithmic concentration–time curves of MRX-8039. (**e**) Concentration–time curves of PMB. (**f**) Semi-logarithmic concentration–time curves of PMB. The green, yellow, blue, and pink lines indicate subcutaneous administration of 2 mg/kg, 4 mg/kg, 6 mg/kg, and 8 mg/kg MRX-8, respectively. The light and dark blue lines indicated subcutaneous administered PMB 4 mg/kg and 8 mg/kg, respectively. The error bars indicate the corresponding standard deviation (*n* = 5). PMB: polymyxin B.

The plasma concentration–time and semi-logarithmic curves for MRX-8 and its metabolite, MRX-8039, as well as PMB following subcutaneous administration are illustrated in Figure 3. The pharmacokinetic parameters obtained from the non-compartmental analysis (NCA) are summarized in Table 2. After administration of 2 mg/kg MRX-8 for 8 h to 24 h, the concentrations of MRX-8 and MRX-8039 approached or even fell below the LLOQ (0.0100 mg/L). Thus, AUC_0–24 h_ (area under the drug concentration–time curve from 0 to 24 h) for MRX-8 and T_1/2_ (terminal half-life), AUC_0–8 h_ (area under the drug concentration–time curve from 0 to 8 h), and AUC_0–24 h_ are not available in Table 2. The C_max_ (maximum concentration) of MRX-8 exhibited a dose-dependent increase, measuring at 2.01 ± 0.18 mg/L, 3.33 ± 0.64 mg/L, 3.73 ± 0.54 mg/L, and 5.09 ± 0.77 mg/L after subcutaneous injections of 2 mg/kg, 4 mg/kg, 6 mg/kg, and 8 mg/kg of MRX-8, respectively. As the dosage escalated from 2 mg/kg to 8 mg/kg, T_max_ (peak time) was shifted from 0.38 ± 0.14 h to 3.25 ± 1.50 h. AUC_0–8 h_ for MRX-8 exhibited a linear proportional relationship with the dosage escalating from 2 mg/kg to 8 mg/kg. For MRX-8039, the C_max_ ranged from 0.03 ± 0.01 mg/L to 0.13 ± 0.05 mg/L across the 2 mg/kg to 8 mg/kg dose groups, and the T_max_ was approximately 1.5 h later than MRX-8. Furthermore, T_1/2_, CL_z_ (total body clearance for extravascular administration), V (volume of distribution), and MRT (mean residence time) had no significant difference for both compounds among the 4 mg/kg, 6 mg/kg, and 8 mg/kg groups (*p* > 0.05). The *in vivo* conversion rate of MRX-8 to MRX-8039 was determined from the ratios of AUC_0–8 h_ and AUC_0–24 h_ under the dosages of 4, 6, and 8 mg/kg, which were 2.47 ± 0.11% and 3.95 ± 0.06%, respectively.

As shown in Figure 3, the absorption of MRX-8 was faster, with a T_max_ of 2~4 h, while the absorption of PMB was relatively slow, with a T_max_ of 4~6 h. The AUC_0–8 h_ were 9.55 ± 2.54 mg·h/L and 17.92 ± 3.05 mg·h/L after administrated PMB 4 mg/kg and 8 mg/kg. And AUC_0–24 h_ were 17.09 ± 3.90 mg·h/L and 31.25 ± 3.14 mg·h/L after administrated PMB 4 mg/kg and 8 mg/kg. MRX-8 showed approximately 1.5-fold exposure than PMB after administrated 8 h at the doses of 4 mg/kg and 8 mg/kg, and the exposure of MRX-8 was also 1.3 times higher than PMB at the dose of 8 mg/kg 24 h after administration (Table 2).

### 2.4. Comparative Safety and Nephrotoxicity Evaluations of MRX-8 and PMB in Rats

Upon subcutaneous administration of both drugs, rats exhibited reduced activity, lethargy, irregular breathing, and manifested swelling in the head, nose, and feet. These observed phenomena were likely associated with the histamine release of the rodent-specific polymyxin effect [13,14] and were resolved within 30 min to 1 h after administration.

**Figure 4:**
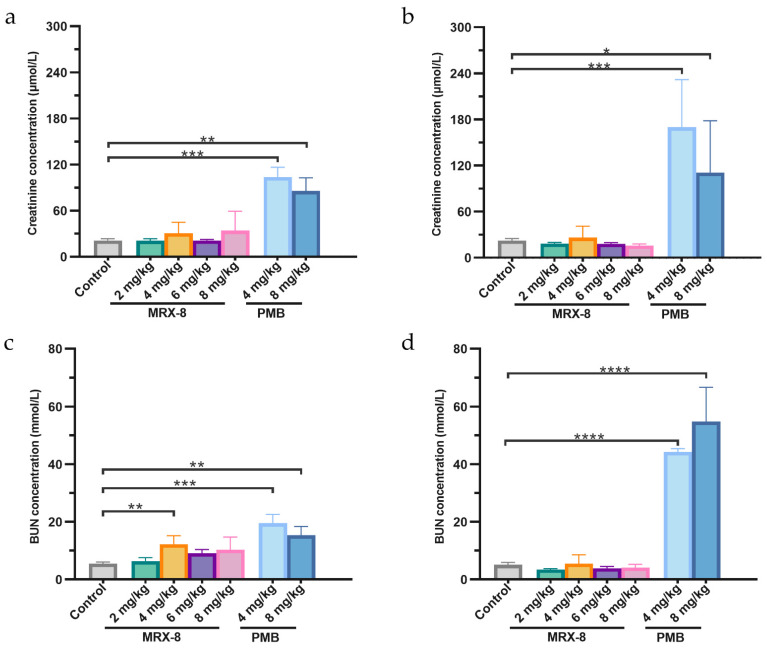
The creatinine and BUN levels after subcutaneous administration of saline, MRX-8, and PMB at 8 h and 24 h. (**a**) Creatinine levels at 8 h. (**b**) Creatinine levels at 24 h. (**c**) BUN levels at 8 h. (**d**) BUN levels at 24 h. Control: saline group; PMB: polymyxin B; BUN: blood urea nitrogen; * *p* < 0.05, ** *p* < 0.01, *** *p* < 0.001, **** *p* < 0.0001.

Creatinine and blood urea nitrogen (BUN) at 8 h and 24 h after administration were evaluated for nephrotoxicity. All the statistics were conducted compared to the saline group. The creatinine concentrations were 21.25 ± 2.22, 30.68 ± 14.26, 34.27 ± 24.97, 103.86 ± 12.66, and 85.61 ± 17.27 μmol/L for saline, MRX-8 4, 8 mg/kg, and PMB 4, 8 mg/kg administration at 8 h, and were 22.5 ± 2.52, 26.28 ± 14.86, 15.77 ± 2.17, 169.79 ± 61.93, and 110.81 ± 67.3 μmol/L at 24 h, respectively. For MRX-8, the blood creatinine levels did not significantly increase at both time points in the dosage range of 2 mg/kg to 8 mg/kg, and the levels at 24 h were slightly lower than at 8 h. In contrast, following the administration of PMB at doses of 4 mg/kg and 8 mg/kg, a significant increase in creatinine levels was observed at both 8 h and 24 h (*p* < 0.05, Figure 4a,b). From the perspective of BUN levels (Figure 4c,d), there was no significant difference for MRX-8 administration, despite a significant increase in 4 mg/kg MRX-8 at 8 h compared to the saline group (12.20 ± 2.99 vs. 5.52 ± 0.56 mmol/L). The level of BUN with MRX-8 administration was also slightly lower at 24 h than at 8 h. Notably, in the case of PMB, at both 4 mg/kg and 8 mg/kg, there was a significant increase in BUN levels at both 8 h and 24 h (19.58 ± 3.05, 15.36 ± 3.05 mmol/L at 8 h, and 44.3 ± 1.12, 54.85 ± 11.81 mmol/L at 24 h), with higher levels at 24 h than at 8 h, indicating that the nephrotoxicity still progressed from 8 h to 24 h post PMB administration.

**Figure 5:**
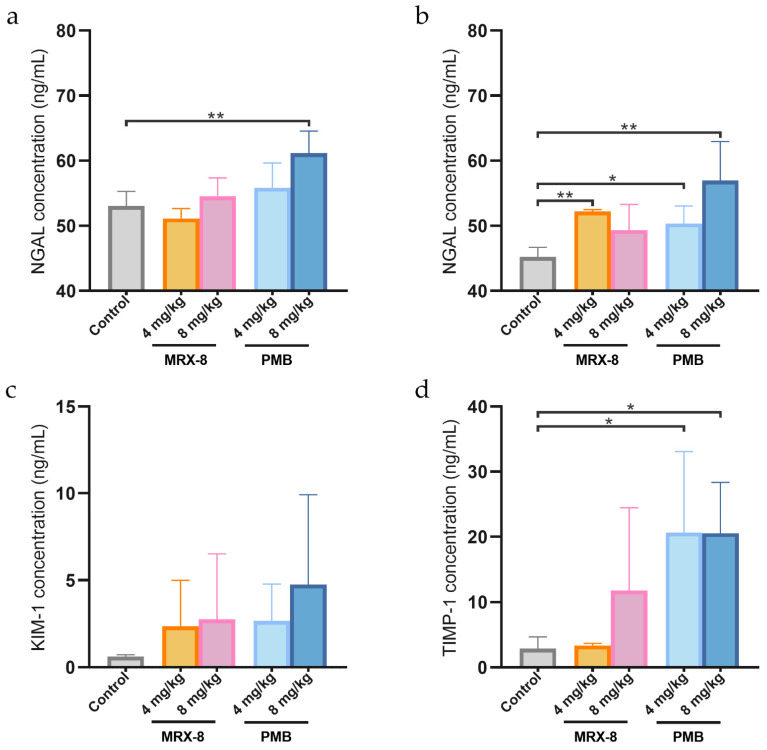
The NGAL levels in plasma and NGAL, KIM-1, and TIMP-1 levels in urine at 24 h after subcutaneous administration of saline, MRX-8, and PMB of 4 mg/kg and 8 mg/kg. (**a**) NGAL levels in plasma; (**b**) NGAL levels in urine; (**c**) KIM-1 levels in urine; (**d**) TIMP-1 levels in urine. NGAL: neutrophil gelatinase-associated lipocalin; KIM-1: kidney injury molecule-1; TIMP-1: tissue inhibitor of metalloproteinase-1; control: saline group, PMB: polymyxin B; * *p* < 0.05, ** *p* < 0.01.

The kidney injury biomarkers of plasma or urinary neutrophil gelatinase-associated lipocalin (NGAL), urinary kidney injury molecule-1 (KIM-1), and urinary tissue inhibitor of metalloproteinase-1 (TIMP-1) levels at 24 h after administration are shown in Figure 5. Among the biomarkers, plasma and urinary NGAL had lower intragroup variability, whereas urinary KIM-1 and TIMP-1 had greater intra-group differences. The results revealed that, compared to the saline group, only the administration of 8 mg/kg PMB resulted in a significant increase in NGAL levels in plasma after 24 h, with concentrations of 61.14 ± 2.52 ng/mL vs. 53.03 ± 2.25 ng/mL (Figure 5a). The urinary NGAL levels were 45.22 ± 1.46, 52.20 ± 0.28, 49.35 ± 3.92, 50.34 ± 2.70, and 56.95 ± 6.01 ng/mL for saline, MRX-8 4, 8 mg/kg, and PMB 4, 8 mg/kg, respectively, and a significant increase was observed for both MRX-8 (4 mg/kg group) and PMB (4 and 8 mg/kg group) (Figure 5b). No statistically significant differences in the urine KIM-1 levels were observed after MRX-8 and PMB administration due to the high variability within each group (Figure 5c). Urinary TIMP-1 increased significantly after the administration of PMB (20.65 ± 12.44, 20.54 ± 7.82 ng/mL for 4, 8 mg/kg, and 2.89 ± 1.80 ng/mL for the saline group), whereas the increase was not significant after the administration of MRX-8 (Figure 5d).

**Figure 6:**
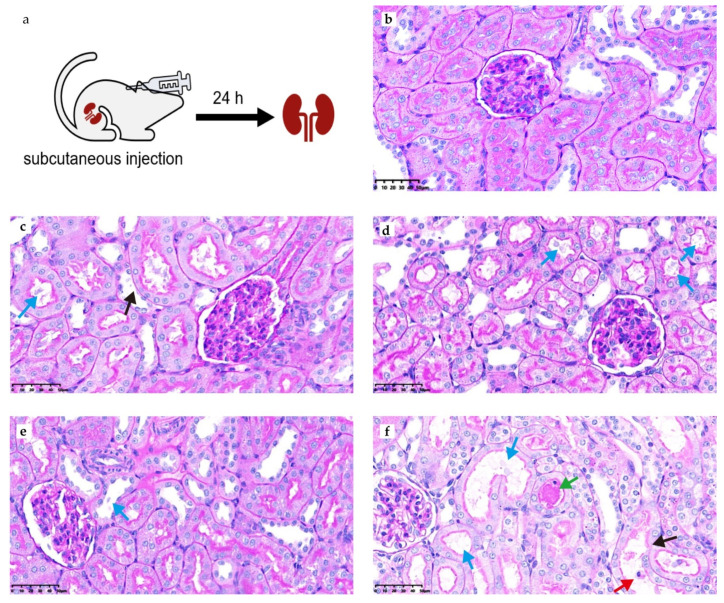
PAS staining of kidney tissue after 24 h of administration with saline, MRX-8, and PMB. (**a**) Experimental design. (**b**) Kidney tissue after saline administration. (**c**) Kidney tissue for MRX-8 at 4 mg/kg. (**d**) Kidney tissue for PMB at 4 mg/kg. (**e**) Kidney tissue for MRX-8 at 8 mg/kg. (**f**) Kidney tissue for PMB at 8 mg/kg. Blue arrows: brush border loss; black arrows: basal membrane exposure; red arrows: nuclear shrinkage; green arrows: tubular formation.

Histological examination of rat kidney tissues at 24 h post the single dose of MRX-8 or PMB administration revealed signs of kidney injury compared to the saline group. MRX-8 and PMB administration resulted in mild focal tubular cell shedding and cytoplasmic attenuation. However, PMB at 8 mg/kg caused more severe renal damage, including tubular dilation, cellular necrosis, and tubular formation (Figure 6). Semi-quantitative scores (SQS), conducted on kidney tissue sections from five rats in each parallel group, are presented in Table 3. After single-dose administration of 4 and 8 mg/kg MRX-8 or PMB, the kidneys showed no significant change, mild or mild-to-moderate renal injury. Compared to the saline group, the chi-square test showed *p* = 0.048 for MRX-8 4 mg/kg and 8 mg/kg, while *p* = 0.008 for PMB 4 mg/kg and 8 mg/kg. The pathological results were consistent with creatinine, BUN, and biomarkers, which all indicated that MRX-8 has lower nephrotoxicity than PMB.

## 3. Discussion

We have developed a reliable and rapid LC-MS/MS method for the simultaneous determination of both MRX-8 and its metabolite, MRX-8039. The established method was difficult due to the differing polarities of MRX-8 and MRX-8039. For ethical and animal welfare reasons, the volume of each plasma sample was reduced to 40 μL from a common 100 μL or 200 μL sampling [15,16]. The method sensitivity was still satisfactory, and there was non-interference from the plasma matrix. The analytical method meets the requirements for the PK analysis toward determining the concentrations of MRX-8 and MRX-8039 in plasma in rats.

The T_max_ of MRX-8 in plasma after subcutaneous administration in rats was delayed at higher doses, probably due to the longer absorption time required for large amounts of the administered agent [17]. The half-life of polymyxins may be species-related [18,19]. Lepak et al. reported that the MRX-8 half-lives in plasma following subcutaneous administration of mice at 0.156 mg/kg, 0.625 mg/kg, 2.5 mg/kg, and 10 mg/kg were 0.53 h, 0.68 h, 0.70 h, and 0.78 h, respectively [8]. Our study has revealed that the half-life of MRX-8 in rats was longer than in mice. And we also reported that the conversion of metabolite MRX-8039 *in vivo* was approximately 4% of MRX-8, as calculated from respective AUC_0–24 h_ ratios.

Notably, the assessment of creatinine and BUN levels revealed an escalating trend 24 h post 4 and 8 mg/kg PMB administration compared to the 8-h time point, suggesting an ongoing trajectory of kidney injury, increasing over time for PMB. In contrast, in rats treated with 4 and 8 mg/kg MRX-8, creatinine and BUN levels at 24 h were lower than those at 8 h, which is indicative of a potential transition into the recovery phase from acute kidney injury. This aligns with the experimental observations of an expedited resolution of respiratory depression adverse effects following the administration of MRX-8 compared to PMB. Consequently, it can be inferred that the adverse effects associated with MRX-8 manifest earlier and resolve more swiftly, along with a potentially milder impact compared to PMB.

Currently, the biomarkers for AKI include NGAL, KIM-1, TIMP-1, liver-type fatty acid-binding protein (L-FABP), interleukin-18 (IL-18), insulin-like growth factor-binding protein 7 (IGFBP7), and others [20,21]. In this study, NGAL, KIM-1, and TIMP-1 were assessed. Among the three biomarkers, NGAL showed the least variation within groups, indicating greater stability. Since urinary NGAL increases may be misinterpreted due to urinary tract infections [22], plasma NGAL was also evaluated to prevent confusion. Kidney injury prompts the release of NGAL from renal epithelial cells, followed by its reabsorption by proximal tubules, and decreased tubular reabsorption after AKI could amplify urinary NGAL levels [23]. This underscored NGAL’s suitability as a biomarker in our study. Conversely, the intra-group variation of the other two urine biomarkers was substantial, although concentrations of both biomarkers increased following the administration of both PMB and MRX-8.

In some studies, PMB was reported to exhibit a comparatively lower nephrotoxicity profile in comparison to colistimethate [24,25]. Thus, using PMB as a comparator in this study serves to further highlight the lower renal toxicity of the MRX-8 advantage. A histopathological study was conducted on kidney tissues from the MRX-8 and PMB groups, and distinctive injury features indicative of PMB-induced AKI were observed, which is in accordance with earlier observations [26,27]. Noteworthy findings included prominent vacuolation of tubular lumens, exposed basement membranes, and crumpling of nuclei. In comparison to the saline group, the 8 mg/kg PMB treatment group exhibited a significant difference in SQS scores (*p =* 0.008), whereas a decreased variance was observed in the 8 mg/kg MRX-8 treatment group (*p* = 0.048).

One study in rats showed that polymyxin=-induced nephrotoxicity was related to the frequency of dosing [28]. A 10-year cohort study found that nephrotoxicity is related to the dose and the number of days of administration [29]. In this study, the drug exposure of MRX-8 was approximately 1.5-fold and 1.3-fold higher than PMB at the dose of 8 mg/kg, while MRX-8 showed lower nephrotoxicity than PMB at the same dose. This supports the use of MRX-8 clinically with a larger, safer therapeutic window. Furthermore, Lepak et al.’s study [8] found that MRX-8 has better antibacterial activity in mice compared to PMB to ensure the effectiveness of MRX-8. Combining the results of both studies on safety and effectiveness, it can be concluded that MRX-8 has good potential for development. However, this experiment was limited to a single dose in rats, and the safety profiles of continuous dosing need further evaluation.

## 4. Materials and Methods

### 4.1. Chemicals

MRX-8 (supplied by MicuRx Pharmaceuticals Co., Ltd., Shanghai, China, with a free base purity of 86.5%) was used as both a reference standard and an administration drug. The metabolite MRX-8039 (with a free base purity of 79.3%) and the IS polymyxin B1 (purity 92.6%) were supplied by MicuRx Pharmaceuticals Co., Ltd. (Shanghai, China) and used for concentration determination. PMB (USP, 1 mg PMB contained 0.734 mg polymyxin B1, 0.086 mg B1-Ile, and 0.090 mg polymyxin B2) was used as an administration drug in rats.

### 4.2. LC-MS/MS Determination Method and Validation

#### 4.2.1. LC-MS/MS Conditions

A Shimadzu LC-30A UHPLC system (Shimadzu, Kyoto, Japan) coupled with a Sciex QTRAP-5500 tandem mass spectrometer (Sciex, Framingham, MA, USA) was utilized. The chromatography was separated on a PHENOMENEX Kinetex^®^XB-C18 column (Phenomenex, Torrance, CA, USA) (100 mm × 2.1 mm, 2.6 μm) at 40 °C. The mobile phases were 2 mM of an ammonium acetate solution with 0.1% formic acid as the aqueous phase and acetonitrile solution with 0.1% formic acid as the organic phase. The gradient elution condition settings are detailed in Table S1. The mass spectrometer was operated in positive ESI mode with the following parameters: nebulizer gas (45 psi), heater gas (55 psi), curtain gas (40 psi), ion-spray voltage 4500 V, and ion source temperature of 450 °C.

#### 4.2.2. Plasma Sample Preparation for LC-MS/MS

An aliquot of 40 μL of rat plasma samples, 50 μL of polymyxin B1 (IS, 20 mg/L), and 50 μL of 6% formic acid in water were mixed. Then, 300 μL of acetonitrile was added to the mixture to precipitate proteins and centrifuged at 4 °C at 13,800 g for 10 min. A 100 μL aliquot of the supernatant was transferred and mixed with 150 μL of 6% formic acid water, and 5 μL was injected into the LC-MS/MS system.

#### 4.2.3. Method Validation

The analytical method was validated following guidelines from the Food and Drug Administration, the European Medicine Agency, and the National Medical Products Administration [30,31,32]. The rat plasma calibration curve ranged from 0.0100 to 10 mg/L for MRX-8 and MRX-8039. Selectivity and specificity were assessed by analyzing six blank rat plasma sources. Calibration curves were required to exhibit linearity (correlation coefficient > 0.98). Inter-day accuracy and precision were assessed with 5 replicates of LLOQ (0.0100 mg/L), QCL (0.0300 mg/L), QCM (0.500 mg/L), and QCH (8.00 mg/L) on the same day. The intra-day comparison involved analyzing three batches over three days, as detailed in a previous study [33]. Precision, calculated by the relative standard deviation percentage, was set at ≤20% for LLOQ and ≤15% for other quality control samples. Accuracy acceptance was within ±15% deviation, except for LLOQ (±20% deviation). Recovery and matrix effects were evaluated in QCL, QCM, and QCH; the relative standard deviation was set at ≤15%. Stability was investigated at various conditions, mimicking the sample collection to analysis.

### 4.3. Pharmacokinetics Experimental Design

A total of 20 wild-type male Sprague-Dawley (SD) rats weighing 280 g ± 20 g were randomly divided into 4 groups. Water and food were adequately provided. Four groups were given MRX-8 at doses of 2 mg/kg, 4 mg/kg, 6 mg/kg, and 8 mg/kg of body weight (dissolved in normal saline). 0.5 mL of blood samples were obtained from the jugular vein (without anesthesia) before and 0.25, 0.5, 1, 2, 4, 6, 8, and 24 h after dosing. Blood samples were collected into tubes with EDTA-K_2_ anticoagulant and centrifuged at 4 °C at 3500 g for 10 min. The plasma was collected and stored in a −70 °C refrigerator until analysis.

In the meantime, 10 additional SD rats were divided into two groups (5 rats each) and administered PMB at 4 mg/kg and 8 mg/kg, separately. Blood samples were obtained before and 0.25, 0.5, 1, 2, 4, 6, 8, 10, 12, and 24 h after dosing. The drug administration and sample collection methods were consistent with those used for MRX-8.

### 4.4. Pharmacokinetics Analysis

MRX-8 and MRX-8039 concentrations in plasma were measured, and mean concentration–time curves were plotted. PK parameters including C_max_, T_max_, T_1/2_, AUC_0–8 h_, AUC_0–24 h_, CL_z_, and V were determined by the NCA module of Phoenix WinNonlin software 8.2 (Certara, Princeton, NJ, USA). After the administration, concentrations below the LLOQ were discarded [34].

### 4.5. Measurement of Kidney Injury Indicators

Rats’ plasma and urine were collected continuously for 24 h after being administered subcutaneously. The plasma collected for the PK assay was split for creatinine and BUN concentrations, whereas a separate experiment was conducted to measure the biomarkers. The concentrations of creatinine and BUN in rat plasma were quantified utilizing the ADVIA Chemistry XPT analyzer (Siemens Healthcare, Munich, Germany). Rat plasma samples, along with quality control samples, were prepared and processed with dedicated reagent cartridges.

Enzyme-linked immunosorbent assay kits specific for KIM-1 (Cell Signaling Technology, Danvers, MA, USA), NGAL (Sangon Biotech, Shanghai, China), and TIMP-1 (Sangon Biotech, Shanghai, China) were utilized for analysis.

### 4.6. PAS Staining and SQS

The kidney tissues were taken 24 h after the PK sample collection. The tissues were embedded in paraffin, sliced, dewaxed into water, and washed for 5 min. We added peracetic acid to oxidize the intracellular polysaccharide glycol group to the dialdehyde group, which combined with the colorless fuchsin in the Schiff reagent and showed violet red, finally deposited on the intracellular polysaccharide. The pathology scores refer to the paper published by Jumana et al. [35]. Briefly, the renal histological assessment included three grades of tubular damage. Scores were assigned to grades: grade 1 = 1, grade 2 = 4, and grade 3 = 10. Kidney involvement was assessed based on the percentage of affected slices: <1% = 0, 1 to <5% = 1, 5 to <10% = 2, 10 to <20% = 3, 20 to <30% = 4, 30 to <40% = 5, and ≥40% = 6. The overall score was calculated by multiplying the percentage score with the grade score. Finally, SQS for renal histological changes was assigned: SQS 0 = no significant change (overall score: <1); SQS +1 = mild damage (overall score: 1 to <15); SQS +2 = mild-to-moderate damage (overall score: 15 to <30); SQS +3 = moderate damage (overall score: 30 to <45); SQS +4 = moderate-to-severe damage (overall score: 45 to <60); and SQS +5 = severe damage (overall score: 60). The samples in this experiment were examined by pathologist Shaojun Liu, who had no knowledge of the grouping.

### 4.7. Statistical Analysis

A one-way ANOVA was performed to evaluate (1) the PK parameters of T_1/2_, CL_Z_, V, and MRT at the dosages of 4, 6, and 8 mg/kg; (2) the concentrations of kidney injury biomarkers between the saline, MRX-8, and PMB groups. For the results of PAS staining, a chi-square test was employed. Due to the sample sizes not exceeding five replicates in each group, Fisher’s exact test was applied accordingly. All statistical analyses were conducted in Graphpad Prism (version 8.0.2) and SPSS (version 25.0); *p* < 0.05 was considered statistically significant.

## 5. Conclusions

In rats, the next-generation polymyxin agent MRX-8 and its primary metabolite MRX-8039 exhibited dose-dependent linearity of PK within 2 to 8 mg/kg doses, with about 4% MRX-8 conversion to MRX-8039 after 24 h. Notably, MRX-8 has demonstrated milder renal toxicity compared to PMB. This key finding has been evidenced by apparently lower creatinine and BUN levels and is supported by the biomarkers of early nephrotoxicity, such as NGAL, as well as the histopathology assessment of kidney tissues. In aggregate, the rat data strongly suggest a reduced kidney injury potential for the new agent MRX-8 in contrast to PMB. The data presented herein support a continued clinical evaluation of MRX-8 for potential use against extensively drug-resistant Gram-negative infections.

## Figures and Tables

**Table 1:** Accuracy and precision for MRX-8 and MRX-8039 in rat plasma matrix.

			MRX-8			MRX-8039		
			Mean Measured Conc (mg/L)	Accuracy (%)	RSD (%)	Mean Measured Conc (mg/L)	Accuracy (%)	RSD (%)
Intra-day (*n* = 5)	LLOQ	0.0100	0.00974	97.4	7.1	0.0109	109.0	8.0
	QCL	0.0300	0.0287	95.6	4.3	0.0302	100.6	5.8
	QCM	0.500	0.501	100.2	7.4	0.515	102.9	6.7
	QCH	8.00	7.60	95.0	7.9	7.79	97.3	4.2
Inter-day (*n* = 15)	LLOQ	0.0100	0.0101	101.1	7.2	0.0103	103.0	14.2
	QCL	0.0300	0.0286	95.2	7.8	0.0293	97.8	7.3
	QCM	0.500	0.524	104.9	9.4	0.505	100.9	8.6
	QCH	8.00	8.12	100.5	8.2	7.88	98.5	8.3

Conc: concentration; LLOQ: lower limit of quantitation; QCL: quality control with low concentration; QCM: quality control samples with medium concentration; QCH: quality control samples with high concentration; RSD: relative standard deviation.

**Table 2:** Pharmacokinetic parameters of MRX-8, MRX-8039, and polymyxin B.

	Dose (mg/kg)	C_max_ (mg/L)	T_max_ (h)	T_1/2_ (h)	AUC_0–8 h_ (mg·h/L)	AUC_0–24 h_ (mg·h/L)	CL_z_ (L/h/kg)	V (L/kg)	MRT (h)
MRX-8	2	2.01 ± 0.18	0.38 ± 0.14	1.21 ± 0.10	6.16 ± 0.78	/	0.32 ± 0.05	0.56 ± 0.06	2.27 ± 0.37
	4	3.33 ± 0.64	1.60 ± 0.55	2.86 ± 0.54	15.00 ± 2.15	18.70 ± 3.85	0.22 ± 0.04	0.91 ± 0.22	4.04 ± 0.41
	6	3.73 ± 0.54	2.20 ± 1.10	2.97 ± 0.31	19.64 ± 3.13	24.54 ± 5.97	0.20 ± 0.12	0.90 ± 0.54	4.12 ± 0.55
	8	5.09 ± 0.77	3.25 ± 1.50	2.66 ± 0.45	27.81 ± 4.83	41.59 ± 10.44	0.20 ± 0.06	0.75 ± 0.12	4.95 ± 0.65
MRX-8039	2	0.03 ± 0.01	1.75 ± 0.50	/	/	/	/	/	/
	4	0.07 ± 0.02	3.33 ± 1.15	8.38 ± 1.49	0.39 ± 0.09	0.72 ± 0.23	/	/	11.98 ± 2.46
	6	0.10 ± 0.01	4.00 ± 0.00	7.67 ± 2.81	0.49 ± 0.11	0.99 ± 0.42	/	/	10.53 ± 2.57
	8	0.13 ± 0.05	4.50 ± 1.00	6.85 ± 0.37	0.66 ± 0.28	1.63 ± 0.54	/	/	10.13 ± 0.84
PMB	4	1.77 ± 0.51	4.80 ± 1.10	4.55 ± 0.56	9.55 ± 2.54	17.09 ± 3.90	0.24 ± 0.06	1.52 ± 0.27	8.66 ± 0.70
	8	3.15 ± 0.44	6.00 ± 0.00	4.09 ± 0.44	17.92 ± 3.05	31.25 ± 3.14	0.25 ± 0.02	1.49 ± 0.31	8.23 ± 0.71

C_max_: maximum concentration; T_max_: peak time; T_1/2_: terminal half-life; AUC_0–8 h_: area under the drug concentration–time curve from 0 to 8 h; AUC_0–24 h_: area under the drug concentration–time curve from 0 to 24 h; CL_z_: total body clearance for extravascular administration; V: volume of distribution; MRT: mean residence time.

**Table 3:** Histological scores of kidney tissues after administration of MRX-8 and polymyxin B.

Abnormality Grade	Percentage of the Kidney Slice Affected in Individual Rats of Each Group (*n* = 5)				
	Saline Control	MRX-8 4 mg/kg	MRX-8 8 mg/kg	PMB 4 mg/kg	PMB 8 mg/kg
1	0, 0, 0, 0, 0	0, 10, 0, 10, 0	0, 10, 0, 0, 0	0, 5, 0, 0, 25	0, 0, 0, 45, 8
2	0, 0, 0, 0, 0	25, 0, 35, 0, 0	0, 0, 15, 8, 15	15, 0, 35, 25, 0	20, 3, 35, 0, 0
3	0, 0, 0, 0, 0	0, 0, 0, 0, 0	0, 0, 0, 0, 0	0, 0, 0, 0, 0	0, 0, 0, 0, 0
SQS for individual rats	0, 0, 0, 0, 0	+2, +1, +2, +1, 0	0, +1, +1, +1, +1	+1, +1, +2, +1, +1	+2, +1, +2, +1, +1

Grade 1: mild acute tubular damage with tubular dilation, prominent nuclei, and a few pale tubular casts. Grade 2: severe acute tubular damage with necrosis of tubular epithelial cells and numerous tubular casts (acute tubular necrosis). Grade 3: acute cortical necrosis/infarction of tubules and glomeruli with or without papillary necrosis.

## Data Availability

The data presented in this study are available on request from the corresponding author.

## Supplementary Materials

The following supporting information can be downloaded at https://www.mdpi.com/article/10.3390/antibiotics13040354/s1. Figure S1: The product ion mass spectra and potential fracture modes of MRX-8 (A) and MRX-8039 (B); Table S1: Gradient elution procedures in liquid chromatography; Table S2: The extraction recovery and IS normalized matrix effect of MRX-8 and MRX-8039 in rat plasma; Table S3: Stability of MRX-8 and MRX-8039 in rat plasma at different storage conditions.
