# Supplementary material for: Pharmacokinetics and Nephrotoxicity of Polymyxin MRX-8 in Rats: A Novel Agent against Resistant Gram-Negative Bacteria
Source: Antibiotics (Basel). 2024 Apr 12;13(4):354. doi: 10.3390/antibiotics13040354 (PMC11047535; doi:10.3390/antibiotics13040354)
Supplement: Supplementary file 1 [file antibiotics-13-00354-s001.zip › antibiotics-2928794-supplementary.pdf]

Pharmacokinetics and Nephrotoxicity of Polymyxin MRX-8 in Rats: A Novel Agent against Resistant Gram-Negative Bacteria

Xingyi Qu [1,2,3], Chenxue Guo [1,2,3], Shaojun Liu [4], Xin Li [1,2,3], Lin Xi [1,2,3], Xiaofen Liu [1,2,3,*] and Jing Zhang [1,2,3,5,*]

1 Institute of Antibiotics, Huashan Hospital, Fudan University, Shanghai 200040, China; 20111030085@fudan.edu.cn (X.Q.); 20211220020@fudan.edu.cn (C.G.); lixin@huashan.org.cn (X.L.); 22211220011@m.fudan.edu.cn (L.X.)

2 Key Laboratory of Clinical Pharmacology of Antibiotics, Shanghai 200040, China

3 National Health Commission & National Clinical Research Center for Aging and Medicine, Huashan Hospital, Fudan University, Shanghai 200040, China

4 Division of Nephrology, Huashan Hospital, Fudan University, Shanghai 200052, China; liushaojun@fudan.edu.cn (S.L.)

5 Clinical Pharmacology Center, Huashan Hospital, Fudan University, Shanghai 200437, China

* Correspondence: xiaofenliu@fudan.edu.cn (X.L.); zhangj61@fudan.edu.cn (J.Z.); Tel.: +86-21-5288-8190 (J.Z.)

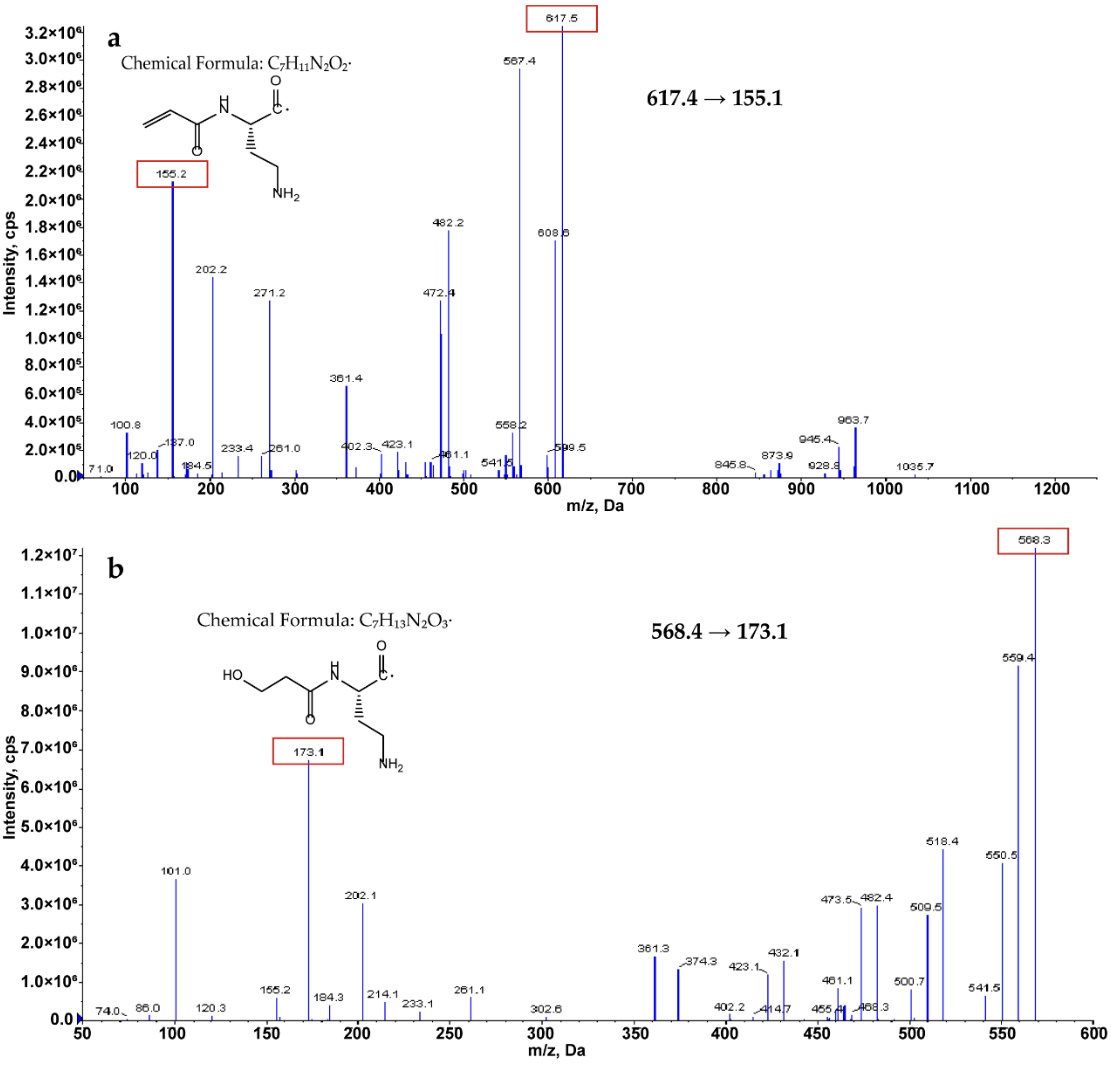

Figure S1. The product ion mass spectra and potential fracture modes of MRX-8 (a) and MRX-8039 (b).

Table S1. Gradient elution procedures in liquid chromatography.

Time (min)	2 mM ammonium acetate solution with 0.1% formic acid	acetonitrile solution with 0.1% formic acid
0	90%	10%
0.3	90%	10%
1.8	35%	65%
2.8	10%	90%
3.3	10%	90%
3.4	90%	10%
4.0	90%	10%

Table S2. The extraction recovery and IS normalized matrix effect of MRX-8 and MRX-8039 in rat plasma.

Samples type*	MRX-8			MRX-8039		
	QCL	QCM	QCH	QCL	QCM	QCH
Concentration (mg/L)	0.03	0.50	8.00	0.03	0.50	8.00
Recovery (%)	87.8±9.1	92.6±7.5	97.7±4.0	85.1±12.2	91.0±9.4	93.9±3.9
Normalized matrix effect (%)	81.2±8.7	94.8±7.3	94.6±2.0	44.5±12.2	50.9±11.8	63.3±8.3

* Six replicas of each quality control sample were quantitated by the calibration curve concentration. The results were expressed as Mean ± RSD. RSD: relative standard deviation. Conc: Concentration.

Table S3. Stability of MRX-8 and MRX-8039 in rat plasma at different storage conditions.

Samples type*	MRX-8		MRX-8039	
	QCL	QCH	QCL	QCH
Concentration (mg/L)	0.03	8	0.03	8
Initial concentrations (%)	101.0±4.6	99.5±6.7	99.1±7.9	96.2±5.2
Freeze-thaw stability (%)	107.3±1.4	104.5±4.5	99.3±11.4	94.3±5.6
Room temperature stability (8 hours) (%)	99.5±6.2	97.3±3.6	96.8±9.9	105.4±3.0
-20◦C stability (42 days) (%)	96.2±9.6	99.9±2.4	92.6±6.7	97.2±6.5
-70◦C stability (42 days) (%)	97.2±6.4	94.2±3.7	107.0±6.0	96.4±2.6

* Three replicas of each quality control sample were quantitated by the calibration curve concentration. The results were expressed as Mean ± RSD. RSD: relative standard deviation.
